# Prevalence of epilepsy and structural brain anomalies in spina bifida aperta

**DOI:** 10.1007/s00381-026-07193-0

**Published:** 2026-03-06

**Authors:** L. R. Koomen, M. Dremmen, C. Kik, R. van den Berg, W. Dronkers, A. J. Eggink, P. L. J. DeKoninck, S. E. M. Veldhuijzen van Zanten, J. Deprest, O. H. J. Eelkman Rooda, S. Koudijs, J. K. H. Spoor

**Affiliations:** 1 Department of Neurosurgery, Erasmus MC University Medical Centre Rotterdam, P.O. Box 2060, 3000 CB Rotterdam, the Netherlands; 2 Department of Radiology and Nuclear Medicine, Erasmus MC University Medical Centre Rotterdam, Rotterdam, the Netherlands; 3 Department of Neurology, Erasmus MC University Medical Centre Rotterdam, Rotterdam, the Netherlands; 4 Department of Obstetrics and Gynaecology, Division of Obstetrics and Fetal Medicine, Erasmus MC University Medical Centre Rotterdam, Rotterdam, the Netherlands; 5 Department of Obstetrics and Gynaecology, University Hospitals Leuven, Leuven, Belgium; 6 Institute of Women’s Health, University College London, London, UK

**Keywords:** Spina bifida, Epilepsy, Nervous system malformations, Arnold- Chiari malformation, MRI, Myelomeningocele

## Abstract

**Introduction:**

Spina bifida aperta (SBA) is frequently associated with a wide range of structural anomalies in the brain, some of these thought to be related to epilepsy. However, the relationship between these is poorly studied. This study aims to assess the prevalence of brain anomalies and epilepsy in SBA patients and explore the nature and magnitude of their association.

**Methods:**

We conducted a retrospective cross-sectional cohort study including all consecutive postnatally treated SBA patients, managed at Erasmus MC Sophia Children’s Hospital, Rotterdam from January 1st 2000 up to June 1st 2018. We determined the presence of structural brain anomalies on magnetic resonance imaging (MRI) and their association using network analysis. The presence of epilepsy was assessed through retrospective evaluation of medical records of patientsWe conducted a retrospective cross-sectional cohort study including all consecutive postnatally treated SBA patients, managed at Erasmus MC Sophia Children’s Hospital, Rotterdam from January 1st 2000 up to June 1st 2018. We determined the presence of structural brain anomalies on magnetic resonance imaging (MRI) and their association using network analysis. The presence of epilepsy was assessed through retrospective evaluation of medical records of patients.

**Results:**

We identified 91 consecutive SBA patients, with a median age of 16.7 years. In 86 patients, the presence or absence of epilepsy could be established; epilepsy was reported in six of them (7.0%). In 75 patients, MRI brain studies were available at a median age of 15 days. Common findings included Chiari malformation type 2 (CMII) (n = 66/74; 89.2%), ventriculomegaly (n = 71/75; 94.7%), corpus callosum dysgenesis (n = 32/70; 45.7%), large massa intermedia (n = 33/75; 44.0%) and hypothalamic adhesions (n = 14/33; 42.4%). Network analysis revealed a dominant cluster of cooccurring anomalies comprising CMII, ventriculomegaly, corpus callosum dysgenesis, and a large massa intermedia.

**Discussion:**

We report on the spectrum of brain anomalies visualized on brain MRI and their cooccurrence patterns in SBA patients. Although the vast majority have structural anomalies, epilepsy was reported in only 7.0% of patients, which is lower than previously reported.

## Introduction

Spina bifida aperta (SBA) is a common congenital abnormality, occurring in approximately 0.51 per 1000 births [1]. Among SBA subtypes, meningomyelocele (MMC) and myeloschisis (MS) are the most frequent [2, 3]. SBA is associated with significant disabilities, including hydrocephalus, pelvic floor dysfunction, and a broad range of neurological deficits below the anatomical level of the spinal abnormality [2, 4].

Fetal ultrasound is a valuable tool for diagnosing SBA and identifying associated anomalies during pregnancy [2]. Early diagnosis and comprehensive assessment are essential for counseling parents regarding prenatal management options. Today the latter includes prenatal surgery of SBA, which has been shown to reduce the need for postnatal shunt placement and to improve motor and urological outcomes [5, 6]. However, prenatal surgery does not cure the condition, and lifelong multidisciplinary care remains mandatory [5].

Fetal ultrasound and magnetic resonance imaging (MRI) often reveal additional brain anomalies with SBA [7, 8], including Chiari malformation type II (CMII) and ventriculomegaly, which are among the most prevalent. CMII in particular is nearly ubiquitous in SBA patients who did not undergo fetal surgery [4]. Other common findings include anomalies of the corpus callosum, which may also contribute to the neurological burden in these individuals [4, 9].

Seizures and epilepsy are frequently present in SBA patients, reported in up to approximately 17% of the cases [10–13]. These rates are substantially higher than in the general population [14], suggesting a predisposition related to the existence of SBA. Some studies suggest a link between hydrocephalus and subsequent shunting and the development of seizures [10, 11, 13]. Structural brain anomalies, such as cortical dysplasia, hippocampal sclerosis, or gray matter heterotopia, are widely considered to play a role in the onset of epilepsy [15–19].

This study aims to determine the prevalence of structural brain anomalies and epilepsy in a single institutional cohort of SBA patients who underwent postnatal repair, and to investigate potential correlations.

## Methods

### Patient population

This retrospective cohort study included all consecutive SBA patients born between January 1 st 2000 up to June 1 st 2018, managed at the Erasmus MC Sophia Children’s Hospital in Rotterdam, the Netherlands, a tertiary referral center for spinal dysraphisms. Inclusion criteria were as follows: (1) patients diagnosed with SBA at any time, (2) who were postnatally managed, and (3) in whom clinical data was available. Patients who did not undergo brain MR imaging were excluded from brain anomaly assessment but were included in the epilepsy assessment. The study was approved by the hospital’s Medical Ethical Committee (MEC-2023-0462).

### Data collection and synthesis

Data on patient characteristics, age at time of MR brain scan, anatomical level of the spinal defect, presence of a shunt and gestational age at birth was collected. Outcomes were collected throughout October 2022 up until March 2023.

MR images were acquired using a 1.5 Tesla General Electric MRI System (GE, Milwaukee, Wisconsin) with an eight-channel head coil. The scanning protocol preferably included a 3D T1-weighted sequence (TR/TE: 8.77/3.4 ms; slice thickness 1.0 mm; matrix 220 × 220) and an axial T2-weighted sequence (TR/TE: 8507/100 ms; slice thickness 2.0 mm, matrix 320 × 320). A pediatric neuroradiologist (*M.D.*) reviewed all available MR brain images to systematically identify the presence of congenital brain anomalies. In patients with multiple MR brain studies, all images were used for analysis. Anomalies were categorized into the following groups as previously described: *cortical and migration abnormalities, deep grey matter abnormalities, midline abnormalities, white matter abnormalities, ventricular abnormalities*,* and infratentorial brain abnormalities* [20, 21]. To assess interobserver reliability, MR images of at least 10% (*n* = 9) of patients, randomly selected, were independently reviewed by a second neuroradiologist (*S.E.M.V.-Z.*). In case of disagreement, the assessment of the primary pediatric neuroradiologist was considered decisive.

Medical records were screened for the presence of (suspected) epilepsy, performed electroencephalograms (EEG), and the use of anti-seizure medication (ASM). Patient data identified using this search strategy were independently reviewed by two neurologists (*S.K., R.B.*) to confirm the diagnosis of epilepsy. The diagnosis of epilepsy was made according to the criteria set by the International League Against Epilepsy [22]: “(1) At least two unprovoked (or reflex) seizures occurring >24 h apart; (2) one unprovoked (or reflex) seizure and a probability of further seizures similar to the general recurrence risk (at least 60%) after two unprovoked seizures, occurring over the next 10 years; (3) diagnosis of an epilepsy syndrome.” To meet criteria (2) and (3), information from available EEGs was used.

### Statistical analysis

Descriptive statistics were calculated using IBM SPSS Statistics (Version 29). For MR images with missing or incomplete data due to quality issues or moving artefacts, only assessable anomalies were included in the analysis. Any anomalies or categories that could not be classified as “present” or “not present” were labeled as “unable to assess” and excluded from further analysis. For anomalies excluded from analysis, the number of patients with assessable data was recalculated to determine the new denominator for that anomaly. Medians and interquartile ranges (IQR) were calculated where appropriate. A *p*-value of <0.05 was considered statistically significant.

The co-occurrence of brain anomalies was visualized using a force-directed graph algorithm. In such graphs, nodes represent the brain anomalies observed, with the size of each node corresponding to the number of observations. The connections between nodes indicate the co-occurrence of two anomalies in the same patient, with the edge thickness representative of the strength of this co-occurrence. This method allows for visual identification of clustering and relationships between anomalies. Graph analysis was performed using the NetworkX Python library [23].

## Results

**Fig. 1:**
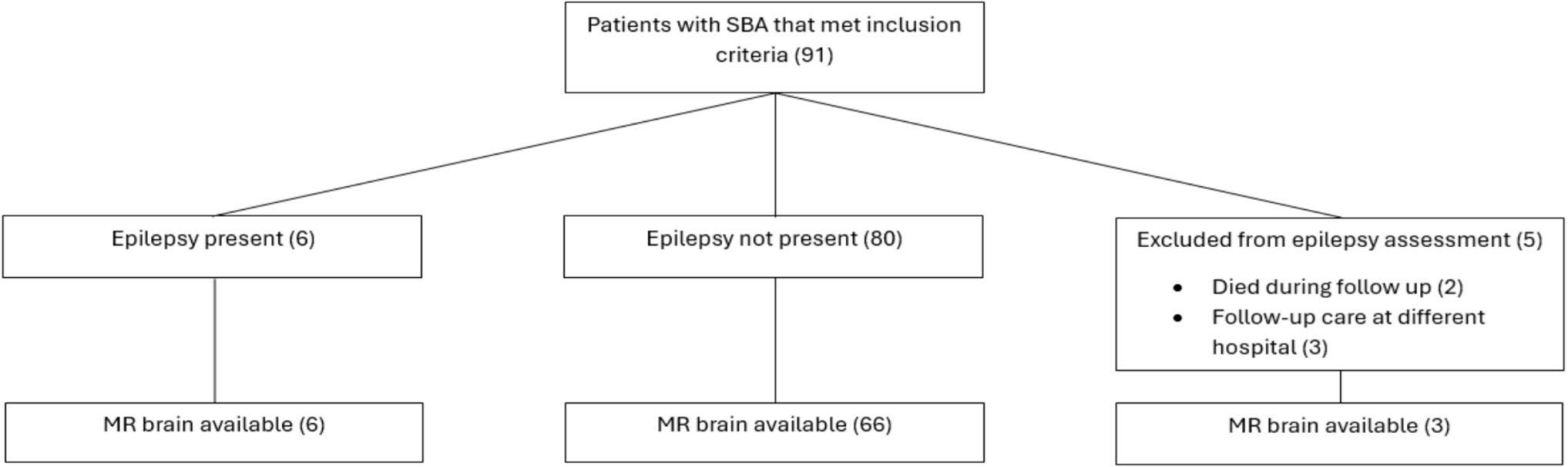
The process of evaluating SBA patients for MRI brain analysis and epilepsy analysis

A total of 91 consecutive SBA patients were reviewed, of whom 75 (82.4%) had MR brain imaging (Table 1). Fifteen patients (18.5%) were born prematurely (<37 weeks). Most SBA defects were located in the lumbar (*n* = 66, 72.5%) region, with fewer SBA defects in the thoracic (*n* = 16, 17.6%) or sacral (*n* = 9, 9.9%) region. Most patients underwent a CSF diversion procedure (*n* = 78, 85.7%), of whom many had repeated surgery (*n* = 64, 70.3%). Age at first CSF diversion procedure ranged from 0 to 116 days (median 11.0 days; IQR 9.0–14.0 days). Median age of patients was 16.7 years (IQR 12.3–19.6) years. Figure 1 displays how SBA patients were evaluated for MRI brain analysis and epilepsy analysis.

**Table 1:** Characteristics of SBA patients

Variables	Total group, *N* = 91 (100%)	Patients included in brain MR imaging assessment, *N* = 75 (100%)
**Sex**		
*Male*	45 (49.5%)	35 (46.7%)
*Female*	46 (50.5%)	40 (53.3%)
**Gestational age at birth**	***N* = 81**	***N* = 70**
<*37 weeks*	15 (18.5%)	13 (18.6%)
≥*37 weeks*	66 (81.5%)	57 (81.4%)
**SBA defect location**		
*Thoracic*	16 (17.6%)	14 (18.7%)
*Lumbar*	66 (72.5%)	53 (70.6%)
*Sacral*	9 (9.9%)	8 (10.7%)
***CSF diversion procedure***	78 (85.7%)	66 (88.0%)
**Brain MR imaging procedure**		
*Within first month of life*	-	49 (65.3%)
*After first month of life*	-	26 (34.7%)

### Brain anomalies

Among 91 SBA patients, sixteen (17.6%) were excluded due to lack of available MR brain images. The age of patients at the time of their first MR brain scan ranged from immediately after birth to 18.7 years (median 15.0 days; IQR 2.0–387.0 days). The majority (*n* = 49, 65.3%) had imaging within the first month after birth. Five premature infants (6.7%) had missing data regarding their exact gestational age at imaging; in all other patients, MR brain imaging was performed at minimum term equivalent age.

A total of 281 structural brain anomalies were identified in 75 patients, with a median of 4.0 (IQR 2.0–5.0) anomalies per patient. Eighteen distinct anomalies were identified. There was only one patient without detectable anomalies. This patient (9.8 years) was born at term with a lumbar SBA, attends regular education, walks without walking aids, has no shunt, yet reports urinary incontinence.

Ventriculomegaly (*n* = 71, 94.7%) and CMII (*n* = 66, 89.2%) were the most frequent anomalies (Table 1). Midline abnormalities such as corpus callosum dysgenesis (*n* = 32, 45.7%), a large massa intermedia (*n *= 33, 44.0%), and hypothalamic adhesions (*n* = 14, 42.4%) were other frequently reported anomalies. Less frequent anomalies included stenogyria (*n* = 6, 9.1%), agenesis (*n* = 11, 14.7%), and dysgenesis (*n* = 9, 12.0%) of the septum pellucidum, dysgenesis of the falx cerebri with interdigitation of gyri (*n *= 6, 8.0%), and a porencephalic cyst (*n* = 6, 8.1%). An overview of every identified brain anomaly can be found in Table 2.

**Table 2:** Overview of brain anomalies in SBA patients, identified on MR brain imaging

Brain anomalies	No. of patients (%)	Epilepsy (%)	No epilepsy (%)
Total	75 (100%)	6 (100%)	66 (100%)^b^
≥ 1 anomaly	74 (98.7%)	6 (100%)	64 (98.5%)
Cortical/migration abnormalities (*n* = 66)^a^			
Heterotopia *Multiple bilateral subependymal heterotopia* *Multiple unilateral subependymal heterotopia* *Solitary unilateral subependymal heterotopia*	15 (22.7%) 8 (12.1%) 1 (1.5%) 6 (9.1%)	1 (20.0%) 0 (-) 1 (20.0%) 0 (-)	13 (22.4%) 7 (12.1%) 0 (-) 6 (10.3%)
Stenogyria	6 (9.1%)	1 (20.0%)	4 (6.9%)
Schizencephaly closed lip	1 (1.5%)	0 (-)	1 (1.7%)
Dysgenesis cingulate gyrus	1 (1.5%)	0 (-)	1 (1.7%)
Midline abnormalities			
Chiari II (*n* = 74)^a^	66 (89.2%)	6 (100%)	57 (86.2%)
Corpus callosum dysgenesis (*n* = 70)^a^	32 (45.7%)	5 (83.30%)	26 (41.9%)
Large massa intermedia	33 (44.0%)	3 (50.0%)	30 (45.5%)
Hypothalamic adhesions (*n* = 33)^a^	14 (42.4%)	3 (100%)	9 (32.1%)
Agenesis septum pellucidum	11 (14.7%)	2 (33.3%)	8 (12.1%)
Dysgenesis septum pellucidum	9 (12.0%)	1 (16.7%)	8 (12.1%)
Dysgenesis falx cerebri with interdigitation of gyri	6 (8.0%)	0 (-)	5 (7.6%)
White matter abnormalities			
Porencephalic cyst (*n* = 74)^a^	6 (8.1%)	0 (-)	5 (7.6%)
Gliosis	1 (1.3%)	0 (-)	1 (1.5%)
Ventricular abnormalities			
Ventriculomegaly	71 (94.7%)	6 (100%)	62 (93.9%)
Septations posterior horn lateral ventricle	2 (2.7%)	1 (16.7%)	1 (1.5%)
Infratentorial brain abnormalities (*n* = 74)^a^			
Dysgenesis of the brain stem	4 (5.4%)	0 (-)	4 (6.2%)
Hypogenesis of the cerebellar tissue	2 (2.7%)	0 (-)	2 (3.1%)
Dysgenesis of the vermis	1 (1.4%)	0 (-)	0 (-)

^a^In some patients presence of this anomaly could not be assessed, creating a subsample of assessable patients

**Table 3:** Characteristics of SBA patients with epilepsy

Patient no.	Seizure type	Age of onset	Seizure control	Location of epileptic focus based on EEG
1	Focal + Generalized	2 years, 6 months	Relatively good	Centrotemporal/frontal left
2	Generalized + Unclassifiable	Possibly neonatal, new seizure at 12 years, 6 months	Good	
3	Generalized	5 years	Good	
4	Focal + Generalized	11 months	Good	Frontal right > left
5	Focal + generalized	12 months	Poor	Left hemisphere
6	Focal otherwise unspecified + unclassifiable	1 years, 8 months and again 6 years, 5 months	Good	Central right > left

Additionally, in four patients (5.3%) MR brain imaging showed hypovolemic and dysplastic cerebellar tonsils, indicating a previously present CMII. In these cases, CMII was considered present in our assessment. Lastly, seven patients (9.3%) were found to have a corpus callosum described as gracile or slender in aspect, but not dysgenic.

### Co-existence of brain anomalies

**Fig. 2:**
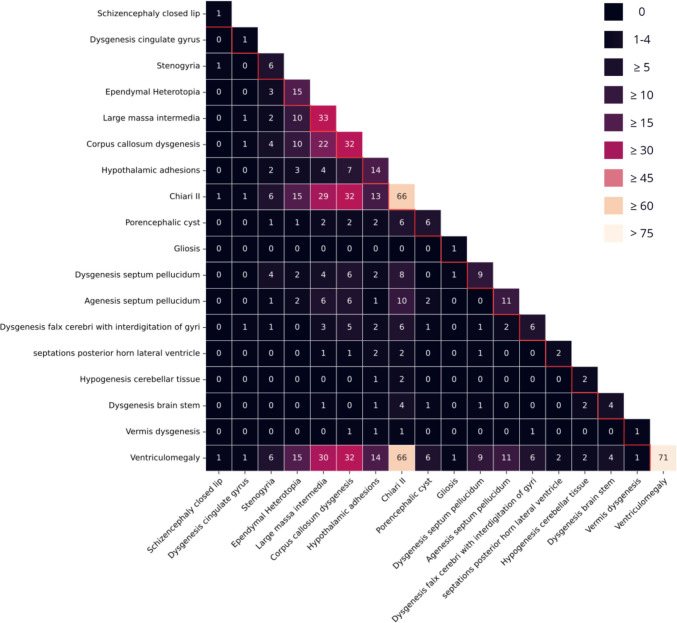
Co-occurrence of 18 MR brain imaging anomalies in 75 SBA patients. Numbers on the diagonal represent the total frequency of each observed brain anomaly in the cohort, while off-diagonal values indicate the frequency with which each pair of anomalies co-occurred

The heatmap (Fig. 2) displays the co-occurrence of brain findings. CMII and ventriculomegaly frequently co-exist with each other, as well as with other anomalies. A large massa intermedia and corpus callosum dysgenesis are also commonly seen together. Other anomalies that often co-occur include stenogyria with dysgenesis of the septum pellucidum, and subependymal heterotopia with either a large massa intermedia or corpus callosum dysgenesis. Additionally, dysgenic falx cerebri frequently co-exists with corpus callosum dysgenesis.

**Fig. 3:**
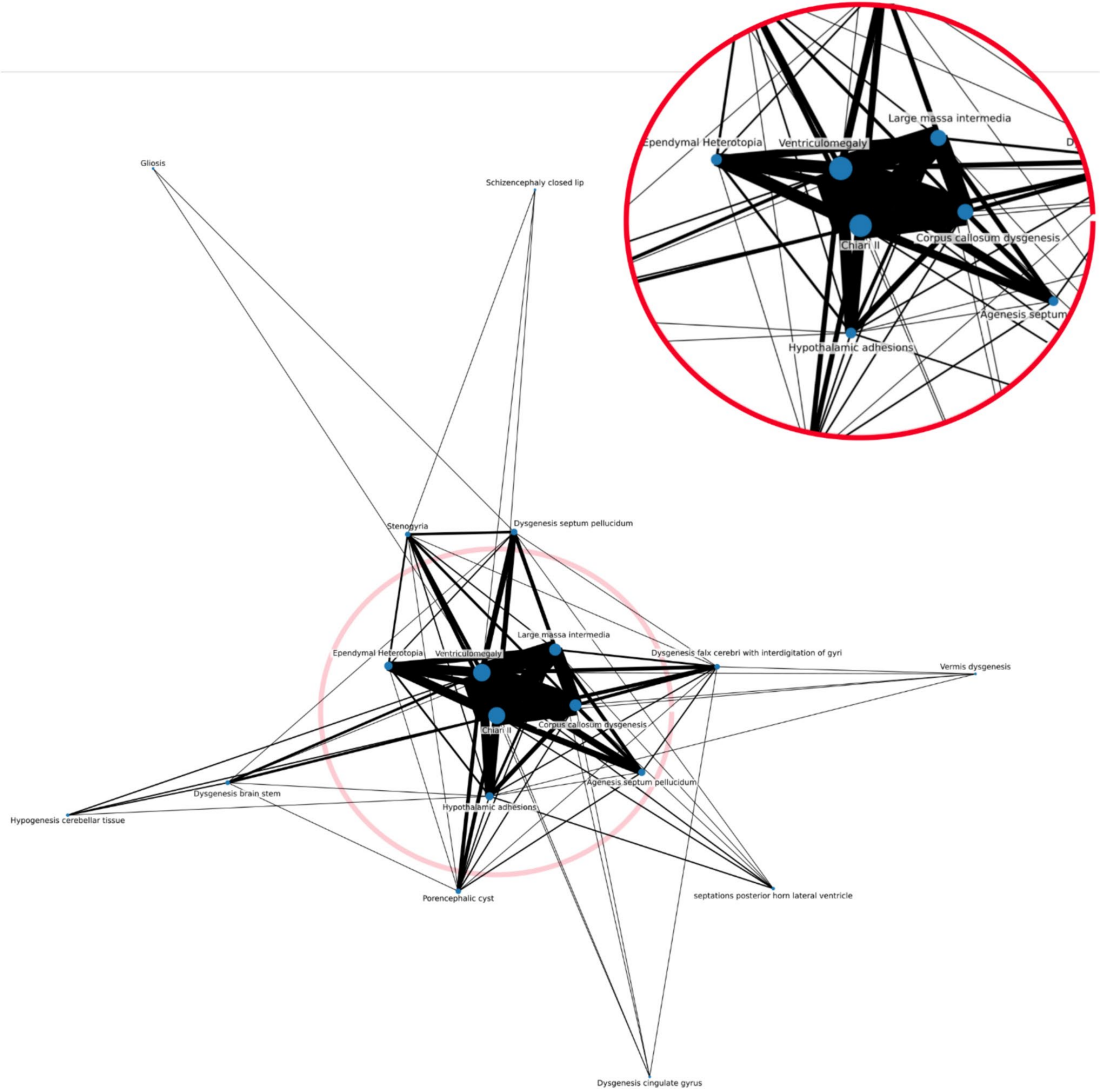
Network analysis of brain anomalies in SBA patients, illustrating the interconnections between different brain anomalies. Central nodes, representing the most frequently co-occurring anomalies, are enlarged for clarity

Figure 3 visualizes a network of identified brain anomalies using a force-directed graph. At the center are the four anomalies (CMII, ventriculomegaly, large massa intermedia, and corpus callosum dysgenesis) that are very frequent, as suggested by the large nodes and thick interlinking edges. This visualizes that these anomalies frequently coincide. While subependymal heterotopia and hypothalamic adhesions are not a part of this central cluster, they visually show a stronger relationship to this cluster than to most of the other anomalies.

### Epilepsy

Among 91 SBA patients, five patients (5.5%) were lost to follow-up before presence or absence of epilepsy was determined in the cohort and were thus excluded; two of these patients died during follow-up and three received follow-up care at different hospitals. Six of the remaining 86 patients (7.0%) had confirmed epilepsy. In all epilepsy patients, MR imaging was available. In five patients with epilepsy, MR imaging was performed *after* the onset of epilepsy, with the interval between first seizure and MR imaging varying. In four patients, the interval was more than one year. An epileptic focus could be identified by EEG in four of six patients. In one patient, epilepsy control was poor; in the other cases it was well controlled with ASM usage (*n* = 4) or with expectant management (*n* = 1).

**Fig. 4:**
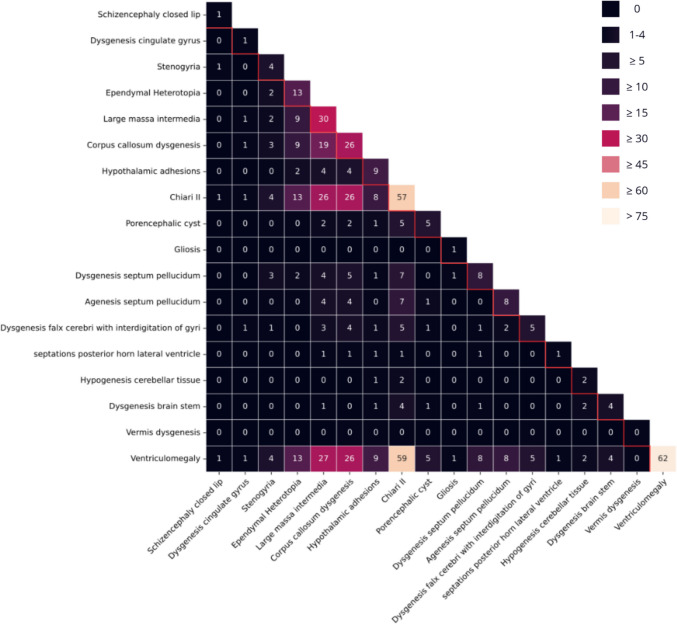
Co-occurrence of 18 MR brain imaging anomalies in 66 SBA patients without epilepsy. Numbers on the diagonal represent the total frequency of each observed brain anomaly in this subgroup, while off-diagonal values indicate the frequency with which each pair of anomalies co-occurred

**Fig. 5:**
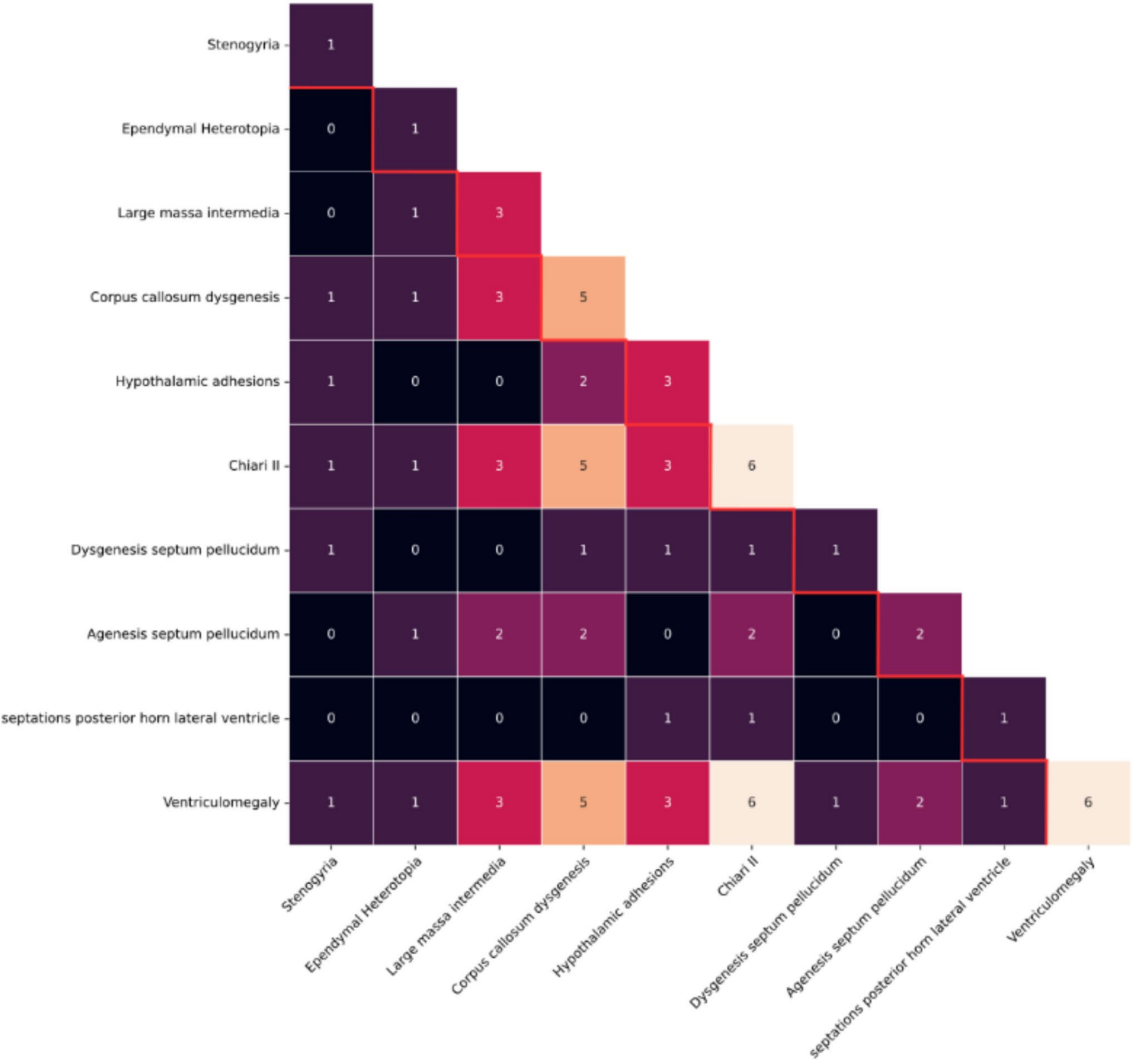
Co-occurrence of 10 MR brain imaging anomalies in six SBA patients with epilepsy. Numbers on the diagonal represent the total frequency of each observed brain anomaly in this subgroup, while off-diagonal values indicate the frequency with which each pair of anomalies co-occurred

In these six epilepsy patients, the number of reported structural anomalies per patient ranged from four to six, with a median of 4.5. All six patients had CMII and ventriculomegaly. Other anomalies included dysgenesis of the corpus callosum (*n* = 5), hypothalamic adhesions (*n* = 3), large massa intermedia (*n* = 3), agenesis of the septum pellucidum (*n* = 2), dysgenesis of the septum pellucidum (*n* = 1), stenogyria (*n* = 1), subependymal heterotopia (*n* = 1), and septations of the posterior horn of the lateral ventricle (*n* = 1). Complete heatmaps of combinations of brain anomalies in epilepsy patients and non-epilepsy patients are included in Figs. 4 and 5. Due to the low number of epilepsy patients, no statistical testing was attempted.

All epilepsy patients had previously undergone one or multiple CSF diversion procedures. Among patients without epilepsy, 84.7% (*n* = 72) had fewer such procedures. No coincidence was observed between CSF diversion procedures and the onset of epilepsy. In all affected patients, epilepsy developed only well after initial CSF procedure (Table 3).

## Discussion

Assessment of the prevalence of brain anomalies in SBA patients operated after birth confirmed that CMII and ventriculomegaly are the most commonly observed brain anomalies. The present study revealed that the common anomalies such as CMII, ventriculomegaly, a large massa intermedia, and corpus callosum dysgenesis frequently co-exist among SBA patients. Finally, we found that the prevalence of epilepsy in our SBA cohort was 7.0%.

Our results are in line with previous studies showing high prevalence rates of ventriculomegaly and CMII in SBA patients. For example, both ventriculomegaly and CMII were observed at similar rates in our cohort, in line with several other studies [24]. These two anomalies are often considered characteristic of the neurological phenotype in children with SBA. Also the high prevalence of corpus callosum anomalies in our study corresponds with that of other studies [11, 24–27], confirming that disruptions in callosal development are common in SBA patients. A reduced corpus callosum thickness, or the so-called gracile corpus callosum, has been suggested to be related to hydrocephalus [28]. We indeed observed that six of the seven patients with a gracile corpus callosum also had ventriculomegaly. A large massa intermedia and septum pellucidum anomalies were often present, nearing the average prevalence of these anomalies in various studies [11, 24–27]. A few patients with porencephalic cysts were observed in this cohort, but in four out of six patients these cysts were deemed iatrogenic as sequelae from previous shunt tracts. This was seen in 5.7% of CSF diversion procedures.

Although various similarities with previous studies were observed, some notable differences were found. We observed a lower prevalence of stenogyria compared to some other studies, which report rates as high as 73% [24, 26]. We presume that this discrepancy might be attributed to differences in the diagnostic criteria used and/or interobserver variability in identifying these subtle cortical malformations. Falx hypoplasia is slightly more frequently reported in the literature as well (20.0% and 24.2% [11, 25]) compared to 8.0% in our cohort. We observed subependymal heterotopia in 22.7%. In the literature, heterotopia ranges from virtually absent till present in a third of SBA patients [11, 24, 26, 27, 29]. This wide range of prevalence may be due to the small sample sizes in those studies, or may indicate that heterotopia can easily be overlooked in the absence of correct MR brain imaging sequences. Polymicrogyria, which was previously occasionally reported in SBA patients [11, 25, 27], was not present in our cohort. Jansen et al. [30] reported incidental brain imaging findings in 3966 children in Rotterdam in their Generation R study. In comparison with their pediatric cohort, reflecting the general pediatric population, anomalies of the corpus callosum, ventriculomegaly, anomalies of the septum pellucidum, or heterotopia are far more present in SBA patients [30].

One anomaly that has received relatively little attention in previous studies is hypothalamic adhesions, which we identified in 42.4% of our patients. Few studies so far have documented such adhesions in SBA patients, and their clinical significance remains poorly understood. Miller et al. [26] reported hypothalamic adhesions in 48.6% of patients with a CMII, while Morais et al. [31] reported a prevalence of 18.9%. While some studies have speculated it could be an atypical form of holoprosencephaly [32] or be related to neuronal migration abnormalities [33], there is limited evidence to support these theories. Our study did not find an obvious association between hypothalamic adhesions and other anomalies, apart from CMII and ventriculomegaly, which are anyway frequent. Importantly, in our study adequate MRI brain sequences for the assessment of hypothalamic adhesions were frequently unavailable. This led to a small subsample of evaluated patients regarding this anomaly, making our estimated prevalence of hypothalamic adhesions susceptible to over- or underestimation. Nonetheless, the clinical implications of hypothalamic adhesions remain unclear and warrant further investigation.

Another important observation is the low prevalence of epilepsy (7.0%), while in some other studies, the prevalence of epilepsy and seizures is as high as 17% [10–13]. This difference may be attributed to several factors, such as differences in the population studied. The relatively low prevalence of epilepsy in our study could also be attributed to the nature of the anomalies in our cohort. While several studies [11, 34] have found severe cortical malformations and neuronal migration disorders in combination with higher rates of epilepsy, we did not observe the same extent of severe brain anomalies in our patients. This may suggest that the overall neurological damage in our cohort was less severe compared to other populations, which could contribute to our relatively low prevalence of epilepsy found in SBA patients.

In our study, all patients affected by epilepsy had undergone CSF diversion procedures. However, we found no evidence to support a direct causal relationship between these procedures and the development of epilepsy. Notably, active ventriculomegaly requiring CSF diversion procedure was considerably more present among these patients. This observation might suggest that increased intracranial pressure in the neonatal brain could contribute to the pathogenesis of epilepsy in this specific patient group. Several researchers proposed the possibility of underlying brain anomalies being causally related to epilepsy [10, 12, 35]. Karakas et al. [11] report a relationship between falx dysgenesis (*p* = 0.004), cortical atrophy (*p* = 0.028) and epilepsy. In our study, cortical atrophy was not observed at all, and in five patients with dysgenesis of the falx cerebri none had epilepsy. The findings from our study rather suggest that specific brain anomalies, while common in SBA patients, do not correlate with the development of epilepsy. This adds to the growing body of evidence that supports the complexity of epilepsy in SBA patients, suggesting that it may result from a combination of genetic, structural, and environmental factors rather than being solely attributable to the presence of brain malformations [36].

There are several limitations to this study. First, the retrospective design meant that some data were incomplete or missing, particularly regarding gestational age and adequate MRI sequences. Additionally, the variability in MR imaging data quality and sequence coverage resulted in the exclusion of some patients or data, potentially biasing our results. Third, we conducted our study on a postnatal cohort, whereas outcomes in fetuses terminated or operated before birth are not included. However, our cohort remains one of the largest studied, and the systematic approach to data collection helps minimize the risk of bias. Future prospective studies with more standardized imaging protocols and larger sample sizes are necessary to confirm our findings and, for instance, explore the clinical significance of hypothalamic adhesions and other anomalies in greater depth.

## Conclusions

This study provides a comprehensive overview of the prevalence and co-occurrence of brain anomalies in postnatally treated SBA patients. The prevalence of epilepsy was low and there was no specific pattern of brain anomalies found that would predict the development of epilepsy.

## Data Availability

Raw data were generated at Erasmus MC Rotterdam. Derived data supporting the findings of this study are available from the corresponding author [J.K.H.S.] in case of a sensible request.
